# Sino-India difference in collectivism and its association with cultural heritage concerning argumentation

**DOI:** 10.3389/fpsyg.2022.1027599

**Published:** 2023-01-12

**Authors:** Xiaopeng Ren, Dongqin Kuai

**Affiliations:** ^1^Institute of Psychology, Chinese Academy of Sciences, Beijing, China; ^2^Department of Psychology, University of Chinese Academy of Sciences, Beijing, China

**Keywords:** holistic thought, compatriotism, nepotism, collectivism, assertiveness, cultural heritage concerning argumentation, China, India

## Abstract

Cross-cultural studies from a global perspective contend that China and India are both collectivistic cultures. However, it remains unclear whether and why China and India differ in their collectivism. This study examines whether the cultural heritage concerning argumentation explains why Chinese people are more collectivistic than Indians. Convenient samples were taken from online surveys (*N*_China_ = 398, *N*_India_ = 418), and 186 participants from the United States were included in the contrast group. In multiple methods conducted here, the Chinese respondents scored higher in holistic thought, compatriotism, nepotism, familism, and self-interdependence than the Indian respondents, while scoring lower in assertiveness and argumentativeness. Although China and India were more collectivistic than the United States, these findings support the hypothesis that Chinese people are more collectivistic than Indians. The study extended our knowledge of individualism–collectivism beyond east–west comparison.

## Introduction

Group-based differences, typically individualism–collectivism (IND-COL), are the core themes of cultural psychology (Oyserman, 2017). Most cross-cultural studies on IND-COL compare Confucian culture in East Asia (China, Japan, and South Korea) and Western protestant culture (Europe and North America). Considering this limitation, scholars have called for studies from other perspectives (Van de Vliert, 2020). Global cultures are diverse, and researchers need to pay more attention to societies beyond the West and East Asia (Krys et al., 2022). Doing so will help clarify the causes of IND-COL differences and verify whether the study results between Eastern and Western cultures also apply on a larger scale. For example, San Martin et al. (2018) proposed that the Arab culture has independent and interdependent characteristics by comparing Arabs, Easterners, and Westerners. Moreover, these cultures are often labeled as collectivistic merely because they differ from the highly individualistic cultures examined in previous studies (Henrich et al., 2010). As such, the diversity of collectivism may be ignored (Brewer and Chen, 2007).

India and China, both of which have a long history and a large population, have often been classified in cross-cultural studies as collectivistic cultures from a global perspective. In addition, both have been compared with Western individualistic cultures (Leung, 1987; Nisbett et al., 2001; Verma and Triandis, 2020). Nevertheless, there is little direct comparison between these two countries; hence, it remains unclear whether they differ in any way, and, if so, what socioecological factors could explain such differences. We explore these questions in this study.

## Sino-India differences in collectivism

Although there is scant literature directly comparing China and India, the results of some multicountry studies show that China may be more collectivistic than India. As shown in Table 1, the individualism score of China (25) is lower than that of India (48) in the IND-COL database of Hofstede (2001). Meanwhile, Oyserman et al. (2002a) conducted a meta-analysis of more than 50 cross-cultural comparison studies with the theme of IND-COL and calculated the individualism and collectivism of other countries according to the degree of their difference from the United States and Canada. The results showed that individualism (collectivism) in China is lower (higher) than that in India. Van de Vliert (2011) also found that the overall in-group favoritism in China (0.51) is higher than that in India (0.34).

**Table 1:** Individualism or collectivism and socioecological factors of China and India.

	Data source	Indicators	China	India	Remark
Individualism/collectivism	Hofstede (2001, p. 215)	Individualism	25	48	A larger value indicates higher individualism.
	Oyserman et al. (2002a, pp. 15–20)	Individualism	0.46	0.29	A larger value indicates lower individualism.
		Collectivism	−0.66	−0.56	A larger absolute value indicates higher collectivism.
	Van de Vliert (2011, pp. 507–508)	In-group favoritism	0.51	0.34	A larger value indicates more in-group favoritism.
Ecological factors	The World Bank (2021a)	Annual *per capita* GDP(US$) (2011–2020)	5,614–10,435	1,458–2,101	
	The World Bank (2021b)	Urban population (% of total population) (2011–2020)	51–61	31–35	
	The United Nations Development Programme (2021) (Human Development Report)	Expected years of schooling (years)	14.0	12.2	
	The World Bank (2021c)	Annual *per capita* GNI, PPP(US$) (2011–2020)	10,200–17,090	4,450–6,930	
	China Meteorological Data Center (2021) and India Meteorological Department (2021)	Harshness of climate	61	37	A larger value indicates a higher level of harshness.
	World Health Organization (2015)	Incidence of infectious diseases per 100,000 people	2.46	8.36	
	Government of India Ministry of Statistics and Programme Implementation (2018) and China National Bureau of Statistics (2020)	Rice planting proportion (%)	18.20%	25.80%	

Despite the findings above suggesting that China may be more collectivistic than India, some doubts remain. Specifically, some of the results were from self-report methods, which have methodological shortcomings compared with scenario methods (Peng et al., 1997). Moreover, the aforementioned studies did not investigate the collectivism differences between China and India but rather examined the correlation between IND-COL and other variables at the national level. Thus, it remains unclear whether the IND-COL differences between China and India are trivial or large enough to be considered.

### Comparing the antecedents of collectivism in China and India

We listed some socioecological factors that may cause differences in collectivism between China and India, and their values are presented in Table 1. We found that these factors cannot explain the abovementioned empirical results. For instance, the modernity theory of IND-COL holds that modernity leads to individualism (Inglehart and Baker, 2000; Greenfield, 2009). China is more developed than India on three critical indicators of modernity: *per capita* GDP, urbanization rate, and education level; nevertheless, previous results suggested that China could be more collectivistic than India, which the modernity theory could not explain. The pathogen prevalence theory of IND-COL argues that the prevalence of infectious diseases can foster a tight-knit society (Fincher et al., 2008). Infectious diseases are less prevalent in China than in India, which does not support China being more collectivistic than India. The rice theory of IND-COL proposes that people in rice-farming regions are more collectivistic than those in wheat-planting regions (Talhelm et al., 2014). Given that the proportion of rice cultivation in China is lower than that in India, it follows that India would be more collectivistic than China; as such, the rice theory also cannot explain the aforementioned conclusion in prior studies. Climate economics theory holds that climate harshness positively correlates with in-group favoritism, and the national wealth negatively correlates with in-group favoritism (Van de Vliert, 2011). The climate harshness in China is higher than that in India, whereas the national wealth of China is more than that of India; thus, we cannot infer which country is more collectivistic. Referring to Van de Vliert’s (2011) theory and using the climate harshness in, and *per capita* GNI of, China and India from 2011 to 2020 (Table 1), we found that the estimated theoretic in-group favoritism is lower in China (0.01–0.18) than in India (0.31–0.33). It is usually suggested that China is less collectivistic than India following climato-economic theory of culture. This result also does not support that China is more collectivistic than India.

In summary, the speculative conclusion based on the abovementioned theories is not consistent with the empirical results of prior studies.

### Sino-India differences in the cultural heritage concerning argumentation

Given that the aforementioned socioecological factors do not support that Chinese people are more collectivistic than Indians, there could be other contributory factors that have not been found. Inspired by Nisbett’s viewpoint that the remarkably different cultural traditions between ancient Greece and ancient China caused the psychological and behavioral differences between Westerners and Chinese (Nisbett et al., 2001), we suggest that the diverse cultural heritage between China and India may have contributed to the Sino-India differences in collectivism.

In India, owing to the high independence of city-states and the wide variety of languages and religions, the tradition of argument and dispute regarding sectarian ideas has always existed along with the long-term coexistence of multiple religions, as exemplified in the following expressions: “We do like to speak,” and “This is not a new habit” (Sen, 2007, p. 3). Inevitably, the cultural heritage concerning argumentation influenced the psychology and behavior of Indians. Why and how did the cultural heritage concerning argumentation influence the psychology and behavior of Indians? First, argumentation requires strict logic, which makes the cognition style of Indians more analytical than holistic. Second, debaters need to show high assertiveness to convince each other and the audience. Third, support and encouragement from the audience to the victors can also reinforce and facilitate a society where people are willing to express and argue. Furthermore, Uchida et al. (2020) proposed that people in one community also share the same psychological/behavioral functions of members who directly engage in ecology-related activities. As the socioecological conditions may affect psychological functions at the macro level through social sharing among individuals, the macro culture also can be shared among community members through social norm creation and transmission, collective activities, and collective memories. It means the argumentation activity can affect not only the people who engage in it but also all the community members and descendants. Altogether, it sounds reasonable that the cultural factor originating from argumentation activity can play one important and continuous role in the cognitive style and social relations of Indians.

However, China is quite different from India in this respect. China has been in a state of political unity for a long time in history. The mainstream cultures, Confucianism and Taoism, have been inherited and recognized broadly by Chinese people; thus, the cultural homogeneity in China is higher than that in India. The Chinese enjoyed racial and cultural homogeneity throughout their history (Stavrianos, 2004). Moreover, both Confucianism and Taoism emphasize harmony in relationships and discourage arguments. This characteristic is also reflected in Confucian and Taoist proverbs such as “In practicing the rules of propriety, harmony is to be prized” in The Analects of Confucius (2018) and “Those who are good do not argue, but those who argue are not good” in Laozi (2015). Therefore, the traditional Chinese culture is considered lacking in debate and argumentation because debate and confrontation are regarded as potential threats to social harmony (Becker, 1986). High cultural homogeneity is conducive to social tightness and collectivism (Triandis, 1995). Influenced by their traditional culture, Chinese people focus more on interpersonal relationships, which is also helpful in forming a highly collectivistic society.

The distinct cultural heritage concerning argumentation between the Chinese people and Indians may have shaped their respective psychological/behavioral characteristics. Lu et al. (2020) found that assertiveness is a mediator of the difference in the proportion of leadership positions between East Asians and South Asians in the United States. Studies have verified that South Asians scored higher than East Asians in assertiveness because South Asian cultures encourage assertiveness in interpersonal communication, while humility is encouraged more in East Asian cultures. Lu’s other study also verified that East Asians have higher ethnic homophily than South Asians by analyzing a survey of students from US law schools (Lu, 2021). Although only the immigrants from China/India in the United States and Chinese/Indian Americans were involved in Lu’s studies, it also suggested that the Chinese people may show more in-group favorite than the Indians.

Hence, we propose that the Chinese are more collectivistic than Indians and that the cultural heritage concerning argumentation could explain the differences. Two studies were included. In study 1, we explored whether or not there are differences in collectivism between Sino-India using multiple self-reported explicit beliefs and scenario tasks. In study 2, more scenario tasks were added to confirm Sino-India differences. Furthermore, the cultural heritage concerning argumentation was also included to examine whether it could explain the differences.

## Study 1

In study 1, we explored whether the Chinese are more collectivistic than Indians with multiple self-report explicit beliefs and scenario tasks.

### Sample

We took a convenient sample through social media, mail, and sample service platforms. The survey questionnaire link/QR code was shared with the participants. Before starting to respond the questionnaire, each participant was required to confirm one consent, which was approved by the Institutional Review Board of the Institute of Psychology, Chinese Academy of Sciences.

The sample comprised 211 Chinese (males = 122, females = 89) from 27 administrative regions and 227 Indians (males = 155, females = 72) from 22 states.

### Variables and their measurement

The variables *compatriotism*, *familism*, *nepotism*, and *holistic thought* were tested in study 1. Considering the possible difference between subgroups, we controlled for the gender and subjective socioeconomic status of the study participants.

### Compatriotism

Compatriotism was measured by one item: “When jobs are scarce, should employers give priority to people of this country over immigrants?” was used here to measure compatriotism on a five-point scale (strongly disagree = 1, strongly agree = 5). This item originated from World Values Survey [WVS] (2021) and had been constructed and validated by Van De Vliert (2011).

### Familism

We applied the same measurement method used by Van De Vliert (2011) to test familism and chose four items from the questionnaire of the GLOBE project of House et al. (2004) (Cronbach’s *α* = 0.77). The items were “In this society, children take pride in the individual accomplishment of their parents”; “In this society, parents take pride in the individual accomplishment of their children”; “In this society, aging parents generally live at home with their children”; and “In this society, children generally live at home with their parents until they get married.” All items used a seven-point scale (strongly disagree = 1, strongly agree = 7).

### Nepotism

Nepotism was measured *via* the loyalty/nepotism task, which examines the differences in attitudes toward friends and strangers (Wang et al., 2011). Four scenarios were given in this task, described as follows:

1. Suppose the participant does business with a friend. As the friend is honest, the participant earns 50% more than what he/she should have earned. Now the participant is required to reward his/her friend with his/her own money.
2. Suppose the participant does business with a friend, and the participant earns 50% less than what he/she should have earned as the friend is dishonest. Now the participant is required to punish the friend with his/her own money.
3. Suppose the participant does business with a stranger. As the stranger is honest, the participant earns 50% more than what he/she should have earned. Now the participant is required to reward the stranger with his/her own money.
4. Suppose the participant does business with a stranger and earns 50% less money than what he/she should have earned as the stranger is dishonest. Now the participant is required to punish the stranger with his/her own money.

A variable connecting reward and punishment is used to measure the participants’ attitudes toward friends and strangers. Suppose “A” represents the “amount to reward friend” minus “amount to punish friend,” and “B” represents the “amount to reward stranger” minus “amount to punish stranger.” Then the difference between “A” and “B” can indicate the difference in participants’ attitudes toward friends and strangers, and “A – B” data were used to measure nepotism, the higher the score, the higher the nepotism (Dong et al., 2019).

### Holistic thought

Holistic thought was measured using the triad task (Ji et al., 2004), in which the participants were presented with sets of pictures and asked to classify these pictures. Three objects (e.g., railway track, train, and car) were included in one set. *Holistic thought* was defined as 100% for a participant classifying all pictures by the internal relationship (train and railway track) and 0% if by the category (train and car).

## Results

We conducted four separate sets of one-way analysis of variance (2 × 2 × 2) with the country (China and India), gender (male and female), and socioeconomic status (high and low) as variance factors to compare the differences in compatriotism, familism, nepotism, and holistic thought between the Chinese and Indian participants.

### Compatriotism

The main effect of the country on compatriotism was significant [*F*_(*1,430*)_ = 6.46, *p* = 0.011, partial *η*^2^ = 0.015]. The Chinese (*M _China_* = 4.14, *SD _China_* = 0.93) participants showed higher compatriotism than the Indian participants (*M _India_* = 3.88, *SD _India_* = 1.00).

### Familism

The main effect of the country on familism was significant [*F*_(*1,430*)_ = 23.07, *p* < 0.001, partial *η*^2^ = 0.051]. The Chinese participants (*M _China_* = 5.64, *SD _China_* = 0.80) were more familistic than the Indian participants (*M _India_* = 5.18, *SD _India_* = 1.10).

### Nepotism

The main effect of the country on nepotism was significant [*F*_(*1,430*)_ = 35.53, *p* < 0.001, partial *η*^2^ = 0.076]. The Chinese participants [*M*_China_ = 3.03, *SD _China_* = 4.52] were more nepotistic than the Indian participants [*M _India_* = 0.68, *SD _India_* = 4.13].

### Holistic thought

The main effect of the country on holistic thought was significant [*F*_(*1,430*)_ = 9.64, *p* = 0.002, partial *η*^2^ = 0.022]. The Chinese participants (*M _China_* = 0.77, *SD _China_* = 0.26) were more holistic than the Indian participants (*M _India_* = 0.71, *SD _India_* = 0.25), and the interaction between country and socioeconomic status was significant [*F*_(*1,430*)_ = 8.14, *p* = 0.005, partial *η*^2^ = 0.019). The independent-sample *t*-test showed that the difference in holistic thought between the Chinese (*M _China_* = 0.78, *SD _China_* = 0.26) and Indian (*M _India_* = 0.77, *SD _India_* = 0.24) participants was not significant for those with high socioeconomic status [*t*_(*301*)_ = 0.38, *p* = 0.701]. For participants with low socioeconomic status, the Chinese sample (*M _China_* = 0.76, *SD _China_* = 0.25) scored significantly larger than the Indian sample (*M _India_* = 0.61, *SD _India_* = 0.25) in holistic thought [*t*_(*133*)_ = 3.48, *p* = 0.001, *d* = 0.60].

**Figure 1:**
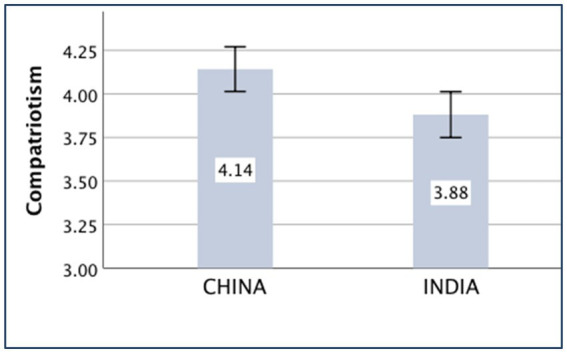
Compatriotism of China and India.

**Figure 2:**
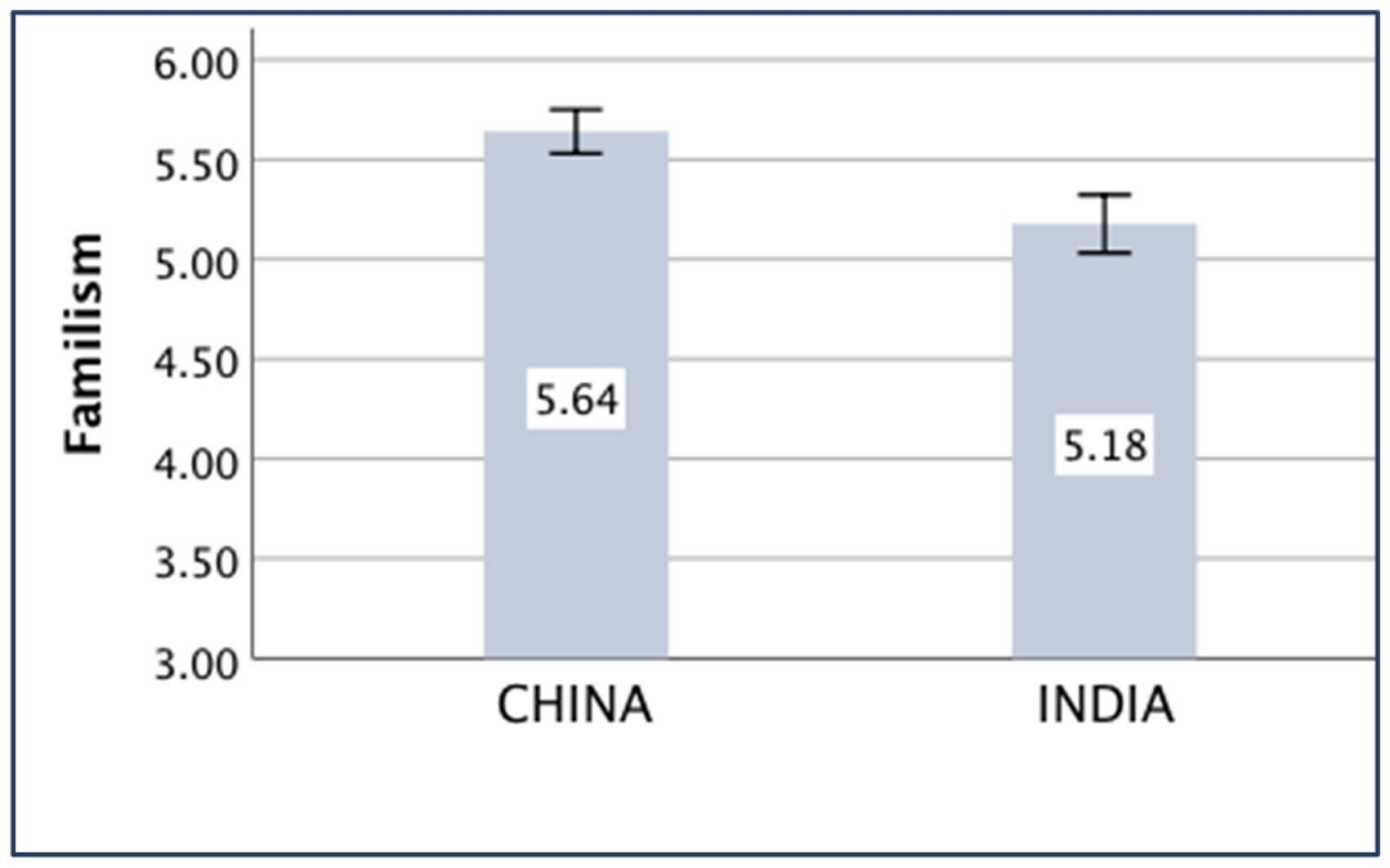
Familism of China and India.

**Figure 3:**
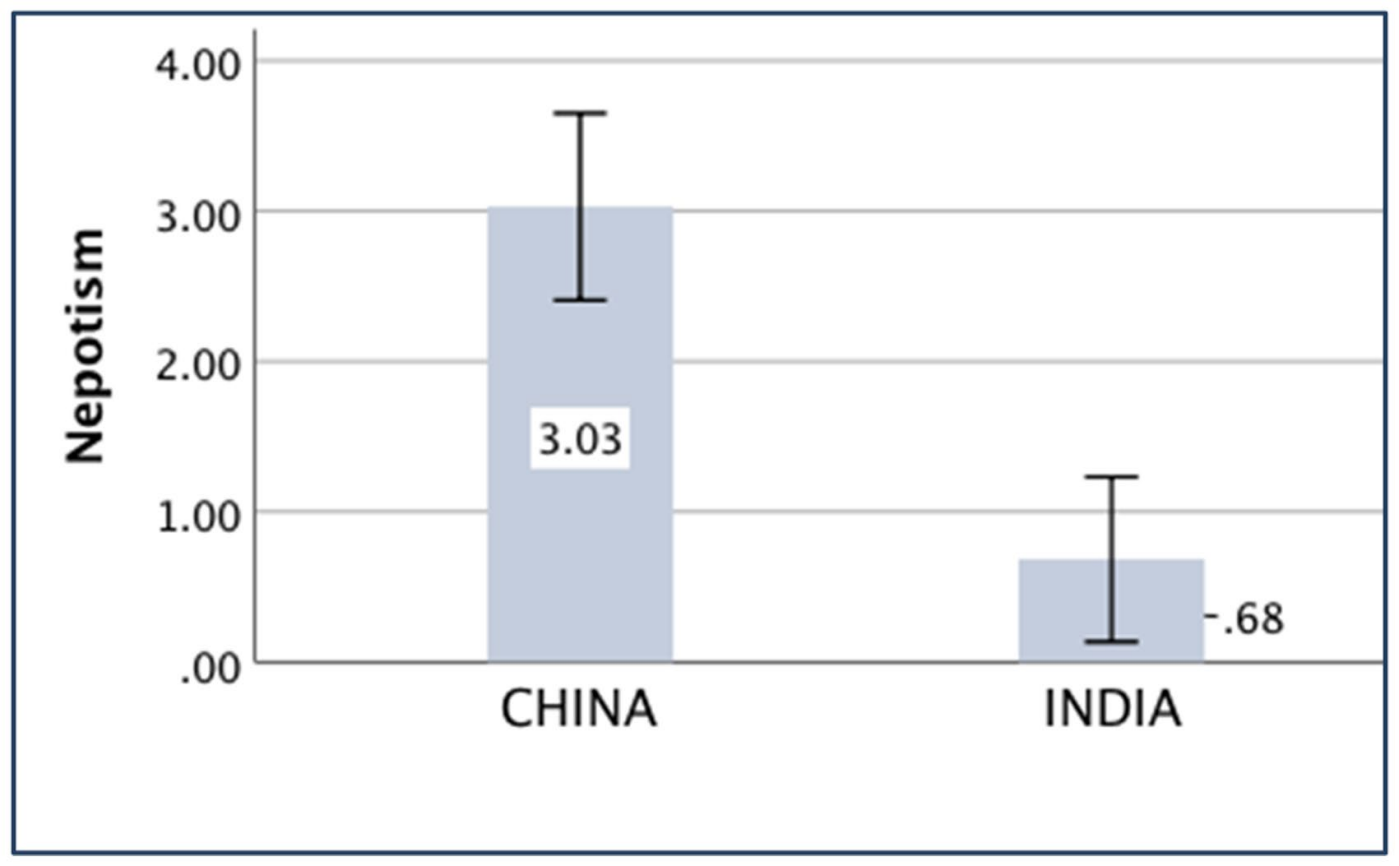
Nepotism of China and India.

**Figure 4:**
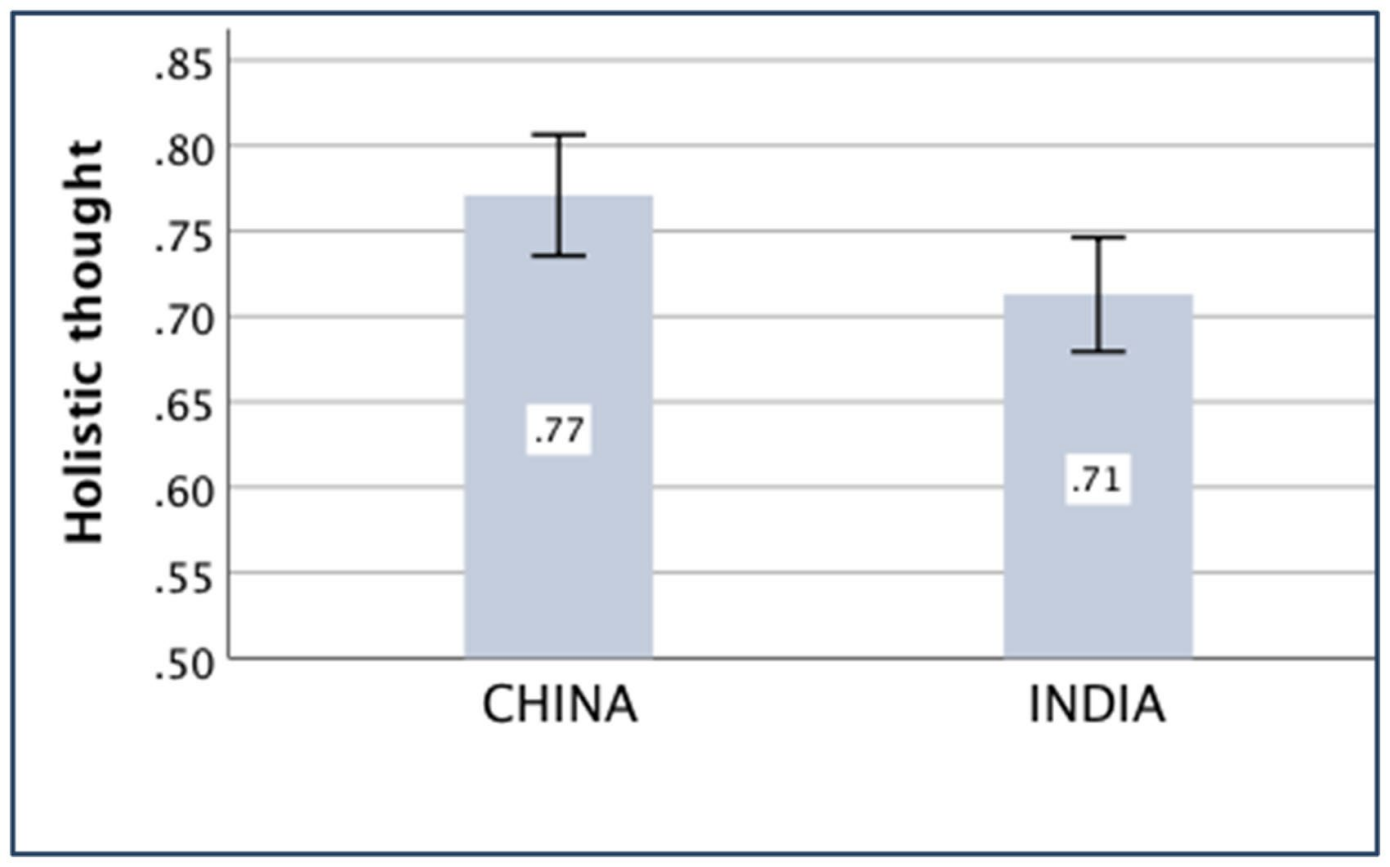
Holistic thought of China and India.

The means of the variables, *compatriotism, familism, nepotism, and holistic thought,* are presented in Figures 1–4.

### Correlations among variables

Some analyses were taken to examine the internal correlations among variables, and the result showed that the correlations are weak or insignificant for both Chinese and Indians. The correlation coefficients among variables are presented in Table 2.

**Table 2:** Descriptive statistics and the internal correlations among the variables for Chinese (below the diagonal)and Indians (above the diagonal).

	*M _China_*	*SD _China_*	1	2	3	4	5	6	*M _India_*	*SD _India_*
1. Gender (male = 0, female = 1)	–	–	–	0.004	−0.093	−0.068	−0.09	0.004	–	–
2. SES (high SES = 1, low SES = 2)	–	–	0.036	–	0.044	0.001	−0.157*	−0.293**	–	–
3. Compatrotism	4.14	0.93	−0.057	−0.013	–	−0.07	−0.105	0.194**	3.88	1.00
4. Familism	5.64	0.80	0.071	−0.004	0.11	–	0.081	0.132*	5.18	1.1
5. Nepotism	3.03	4.52	−0.088	0.026	0.024	−0.029	–	0.208**	0.68	4.13
6. Holistic thought	0.77	0.26	−0.005	−0.063	0.170*	−0.008	0.112	–	0.71	0.25

*p < 0.05, **p < 0.01.

## Discussion

In study 1, it was found that the Chinese were more patriotic, familistic, in-group favorite, and holistic than Indians. It also suggested that modernity, climato-economic, and rice ecology could not explain the Sino-Indian collectivistic differences. It motivated us to continue to explore the potential antecedent of cultural heritage concerning argumentation.

As observed in Table 2, many aspects of collectivism appear to be only loosely connected with behavioral profiles (Na et al., 2020). We confirm the above findings using other measures of collectivism in study 2. Moreover, the cultural heritage concerning argumentation is included to explore whether or not it leads to the differences between China and India.

## Study 2

In study 2, the following three respects were considered. First, collectivism is a multiple-facet or dimensional construct, and we altered some measurements to examine whether or not the findings in study 1 are robust. Second, most measurements of collectivism were developed by east–west comparison. US samples were included to examine the validity of measurements. Third, cultural heritage concerning argumentation was measured to explore whether or not it is one mediator between the culture (China vs. India) and collectivism.

### Sample

Similar to study 1, we took one convenient sample using multiple approaches.

The sample comprised 187 Chinese participants (males = 97, females = 90) from 26 administrative regions and 191 Indian participants (male = 117, females = 74) from 19 states. A total of 186 participants (male = 102, female = 84) are also included from the United States.

### Variables and their measurement

To measure collectivism from other dimensions, the *Inclusion of Other in the Self Scale (IOS)* and the *propensity to experience engaging* vs. *disengaging emotions* were used. In addition, *nepotism*, tested in study 1, was replicated in study 2 to examine the reliability. Furthermore, two other independent scales were used for measuring the assertiveness and argumentativeness of the Chinese and Indian participants. As in study 1, gender and subjective socioeconomic status were controlled for.

### Inclusion of other in the self (IOS) scale

The IOS scale (Aron et al., 1992) is a pictorial measure of closeness using a seven-point scale. Some figures in which the degree of overlap between two circles progresses linearly were presented to participants (test–retest reliability, *r* = 0.83). Participants were asked to select one pair of circles that best represents their relationships with others. Specifically, we asked the participants to select the pair of circles that best represents their mother and one stranger. In a more collectivistic society, the relationship would show greater closeness between participants and in-group members, and less closeness between participants and out-group members (Nisbett, 2004). The *relative IOS*, calculated from the datum of *IOS-stranger* minus *IOS-mother*, was used to measure collectivism.

### Propensity to experience engaging vs. disengaging emotions

We selected two social situations from the Implicit Social Orientation Questionnaire (Kitayama et al., 2006). Only the participant was involved in one of the situations (“watched the TV or listened to music”), and the social relations concerned another situation (“had good interaction with a family member”). Then, we presented the emotions list to the participants. Among these emotions, six different types were embedded: (a) socially disengaging and positive (self-esteem and pride); (b) socially disengaging and negative (frustration and anger); (c) socially engaging and positive (feelings of closeness to others and friendly feelings); (d) socially engaging and negative (shame and guilt); (e) generally positive emotion (elation, happiness, and calmness); and (f) generally negative emotion (unhappiness). Then, the participants were asked to report the extent to which they experienced emotions in each situation, and a six-point scale was used (not at all = 1, very strongly = 6). We calculated the relative intensity of experiencing engaging vs. disengaging emotions as an index of the relative importance of social relations. The score was the mean of *engaging emotions* minus that of *disengaging emotions*. The higher the intensity of experiencing engaging emotions associated with interdependence, the more collectivistic the culture.

### Nepotism

The procedure and test measures for *nepotism* in study 2 were similar to those in study 1.

### Assertiveness

One seven-point scale from Wallen et al. (2017) was used to measure assertiveness (Cronbach’s *α* = 0.85). This scale also was validated by Lu et al. (2020) by comparing South Asian and East Asian assertiveness in Americans. The scale comprised four items. To reduce the influence of the social comparison in the self-judgment process and reduce reference-group effects (Peng et al., 1997; Heine et al., 2002), we asked the participants to evaluate both themselves and the people in their country, and the assertiveness was measured here by the relative value, which used the individual assertiveness minus the population assertiveness in the society as rated by the participant. The participants were asked to indicate how much they agree with each item (strongly disagree = 1, strongly agree = 7). The items were “able to stand own ground in a heated conflict,” “able to use vivid images and compelling logic and facts to support argument,” and “willing to engage in constructive interpersonal confrontations.”

### Argumentativeness

The argumentativeness scale embeds the argument approach (Cronbach’s *α* = 0.91) and argument avoidance (Cronbach’s *α* = 0.86; Infante and Rancer, 1982). The argument approach and argument avoidance were measured here using six selected items from the argumentativeness scale. The argumentativeness trait was calculated using the data by subtracting the total argument avoidance scores from the total argument approach scores. The following were the items used: In China/India, people generally (1) “enjoy avoiding arguments”; (2) “are happy when they keep an argument from happening”; (3) “enjoy a good argument over a controversial issue”; (4) “do not like to miss the opportunity to argue a controversial issue”; (5) “prefer being with people who rarely disagree with them”; and (6) “consider an argument an exciting intellectual challenge.” The tendency to avoid arguments is measured using items 1, 2, and 5; the tendency to argue is measured using items 3, 4, and 6. All items are on a five-point scale (almost never true = 1, almost always true = 5).

## Results

As in study 1, three separate sets of one-way analysis of variance (3 × 2 × 2) with the country (China, India, and the United States), gender (male and female), and socioeconomic status (high and low) as variance factors were conducted to examine the differences in nepotism, IOS, and relative experience of engaging vs. disengaging emotions among the Chinese, Indian, and American participants. The assertiveness and argumentativeness of Chinese vs. Indian participants were compared using another two sets of one-way analysis of variance (2 × 2 × 2) with the country (China and India), gender (male and female), and socioeconomic status (high and low) as variance factors. A mediation analysis was also implemented to examine whether assertiveness is a mediator between a country and another independent variance.

### Inclusion of other in the self scale

The main effect of the country was significant [*F*_(*1,552*)_ = 11.54, *p* < 0.001, partial *η*^2^ = 0.040]. The independent-sample *t*-test showed that the difference between the Chinese (*M _China_* = 3.77, *SD _China_* = 1.73) and American (*M _US_* = 2.79, *SD _US_* = 2.13) participants was significant [*t*_(356)_ = 4.88, *p* < 0.001, *d* = 0.51]. The difference between the Chinese (*M _China_* = 3.77, *SD _China_* = 1.73) and Indian (*M _India_* = 2.96, *SD _India_* = 2.38) participants was also significant [*t*_(347)_ = 3.81, *p* < 0.001, *d* = 0.39], while that between the Indian (*M _India_* = 2.96, *SD _India_* = 2.38) and American (*M _US_* = 2.79, *SD _US_* = 2.13) participants was not significant [*t*_(375)_ = 0.71, *p* = 0.477]. The difference between the Chinese and Americans in IOS showed the cultural validity of the measure. The results revealed that the Chinese are more prone to differentiate between significant others and strangers than Indians.

### Propensity to experience engaging emotions vs. disengaging emotions

The main effect of the country was significant [*F*_(*1,552*)_ = 60.65, *p* < 0.001, partial *η*^2^ = 0.180]. The independent-sample *t*-test showed that the difference between the Chinese (*M _China_* = 1.38, *SD _China_* = 1.15) and American (*M _US_* = 0.63, *SD _US_* = 1.02) participants was significant [*t*_(367)_ = 6.63, *p* < 0.001, *d* = 0.69]. The difference between the Chinese (*M _China_* = 1.38, *SD _China_* = 1.15) and Indian (*M _India_* = 0.19, *SD _India_* = 0.90) participants was significant [*t*_(353)_ = 11.14, *p* < 0.001, *d* = 1.15], while that between the Indian (*M _India_* = 0.19, *SD _India_* = 0.90) and American (*M _US_* = 0.63, *SD _US_* = 1.02) participants was also significant [*t*_(375)_ = −4.41, *p* < 0.001, *d* = 0.46]. The difference between the Chinese and Americans in socially engaging emotion also showed cultural validity. The results revealed that the Chinese feel more socially engaging emotions than Indians.

### Nepotism

The main effect of the country was significant [*F*_(*1,552*)_ = 6.88, *p* = 0.001, partial *η*^2^ = 0.024]. The independent-sample *t*-test showed that the difference between the Chinese (*M _China_* = 2.98, *SD _China_* = 4.34) and American (*M _US_* = 2.11, *SD _US_* = 4.23) participants was significant [*t*_(371)_ = 1.96, *p* = 0.05, *d* = 0.20]. The difference between the Chinese (*M _China_* = 2.98, *SD _China_* = 4.34) and Indian (*M _India_* = 1.32, *SD _India_* = 3.96) participants was significant [*t*_(371)_ = 3.89, *p* < 0.001, *d* = 0.40], while that between the Indian (*M _India_* = 1.32, *SD _India_* = 3.96) and American (*M _US_* = 2.11, *SD _US_* = 4.23) participants was not significant [*t*_(375)_ = 1.88, *p* = 0.061]. The cultural validity of nepotism also was verified by the Sino-US difference. The results indicated that the Chinese are more in-group favorite than Indians.

### Assertiveness

The main effect of the country was significant [*F*_(*1,370*)_ = 10.47, *p* = 0.001, partial *η*^2^ = 0.028]. The Chinese people (*M _China_* = 0.12, *SD _China_* = 0.78) are less assertive than the Indians (*M _India_* = 0.43, *SD _India_* = 1.05).

### Argumentativeness

The main effect of the country was significant [*F*_(*1,370*)_ = 6.55, *p* = 0.011, partial *η*^2^ = 0.017]. The Chinese participants (*M _China_* = −0.16, *SD _China_* = 1.00) reported significantly lower argumentativeness than the Indian participants (*M _India_* = 0.10, *SD _India_* = 0.88).

**Figure 5:**
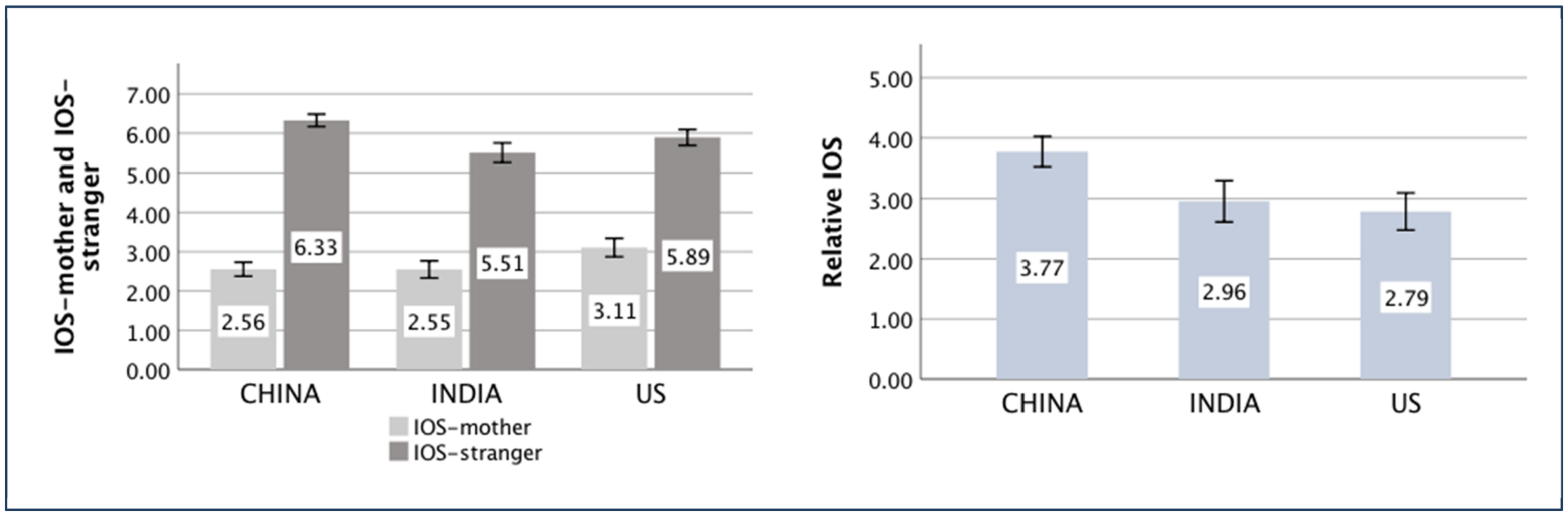
Inclusion of Other in the Self Scale of China, India, and the United States (US).

**Figure 6:**
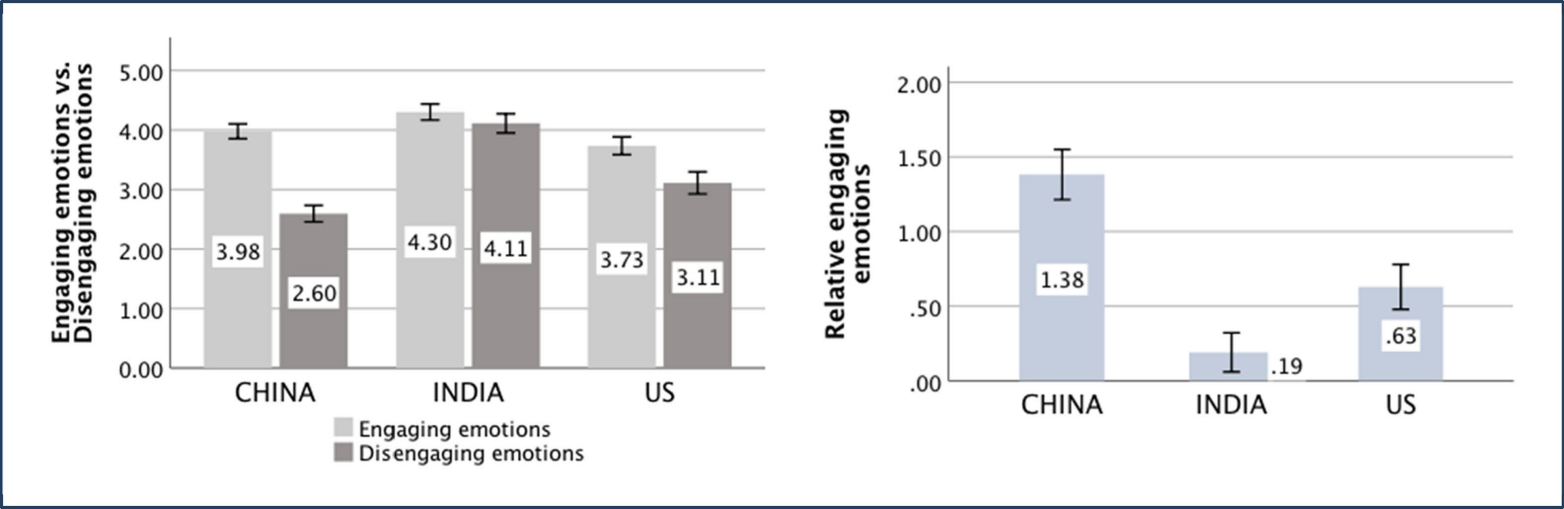
Engaging emotions vs. disengaging emotions of China, India, and the United States (US).

**Figure 7:**
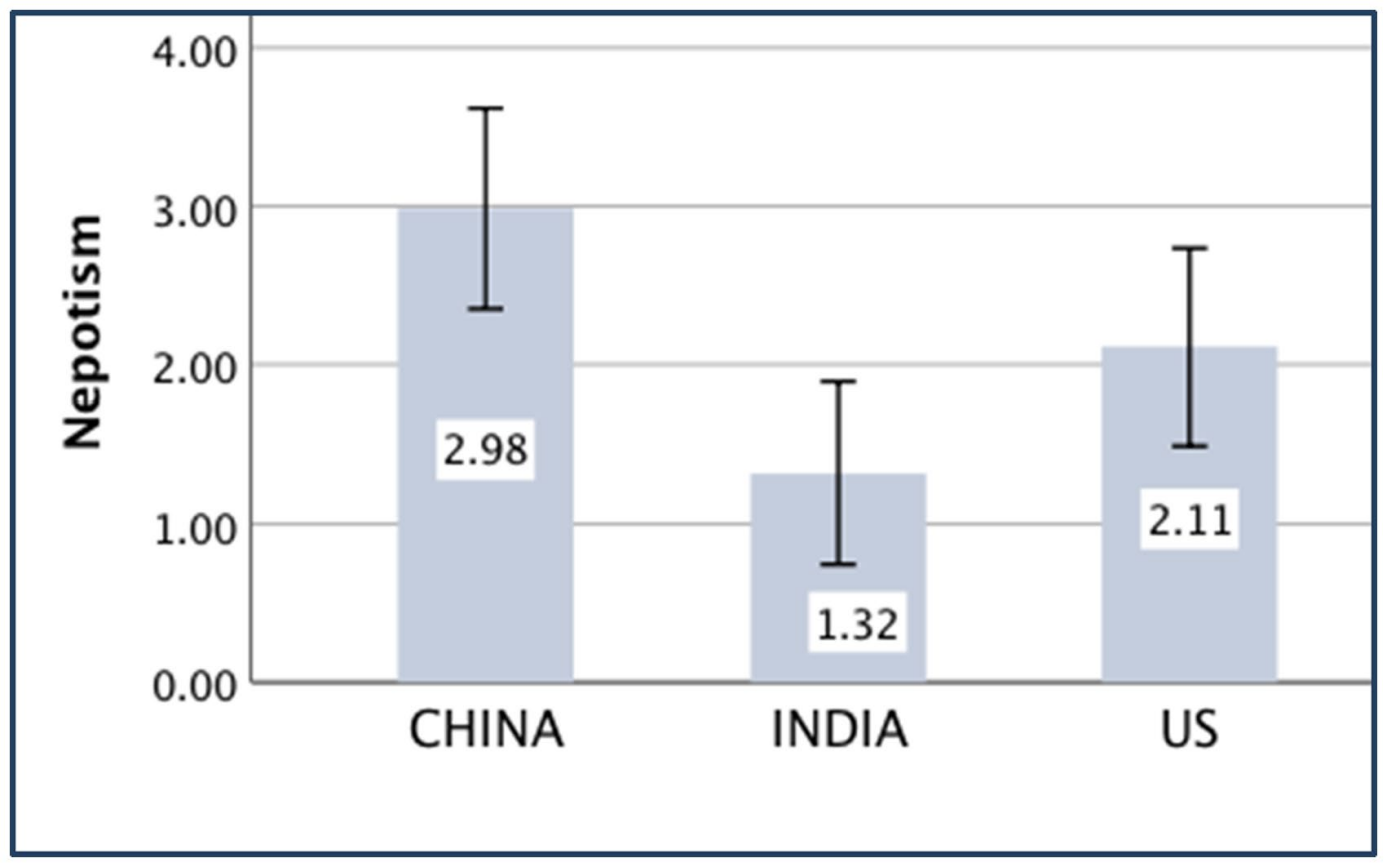
Nepotism of China, India, and the United States (US).

**Figure 8:**
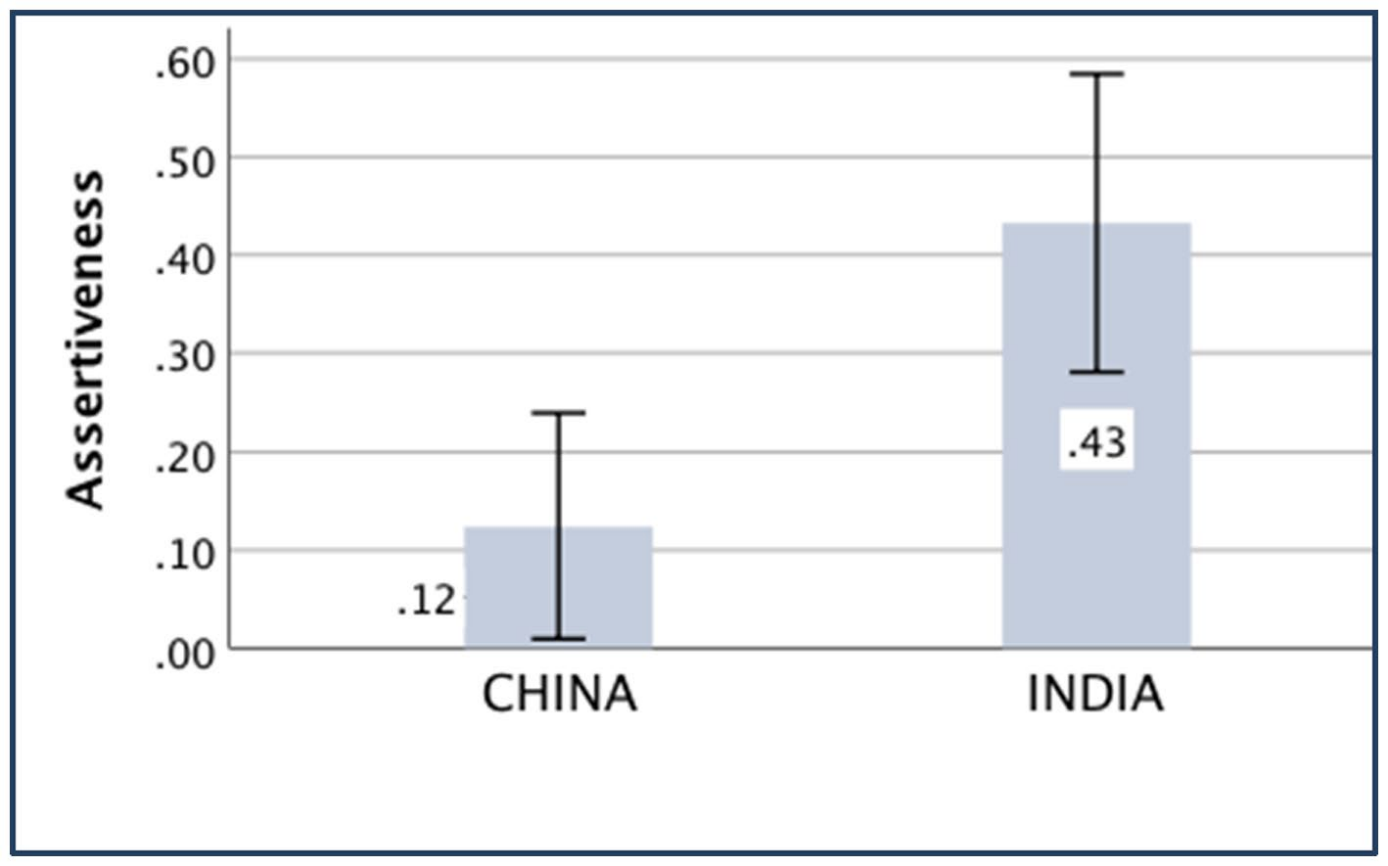
Assertiveness of China and India.

**Figure 9:**
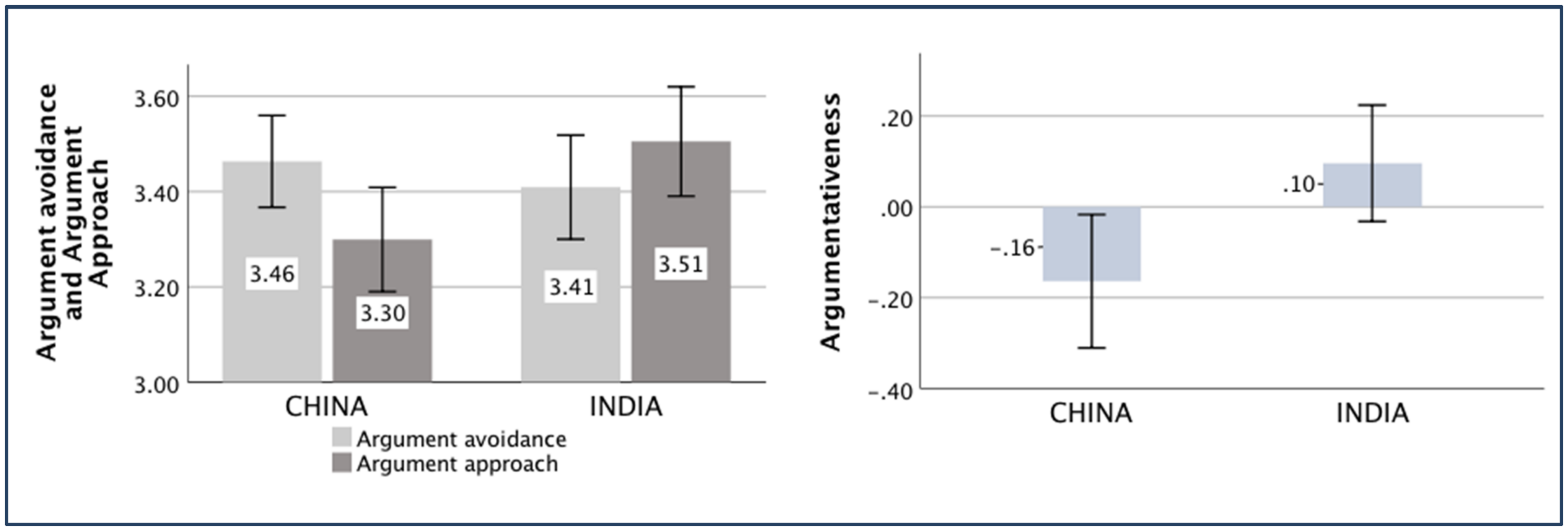
Argumentativeness of China, India.

The means of the aforementioned independent variances are displayed in Figures 5–9.

## Correlations among variables

As in study 1, some analyses also were taken to examine the internal correlations among these variables. The result also showed that the correlations among independent variables are weak or insignificant for Chinese and Indian samples. The correlation coefficients among variables are presented in Table 3.

**Table 3:** Descriptive statistics and the internal correlations among the variables for Chinese (below the diagonal) and Indians (above the diagonal).

	*M _China_*	*SD _China_*	1	2	3	4	5	6	7	*M _India_*	*SD _India_*
1. Gender (male = 0, female = 1)	–	–	–	0.025	0.002	0.084	0.021	−0.074	0.01	–	–
2. SES (high SES = 1, low SES = 2)	–	–	0.046	–	−0.128	0.046	−0.04	0.001	0.004	–	–
3. Nepotism	2.98	4.34	−0.138	−0.013	–	−0.006	0.160*	0.164*	0.01	2.11	4.23
4. Relative engaging emotions	1.38	1.15	0.075	−0.013	0.08	–	0.217**	−0.037	−0.04	0.19	0.90
5. IOS	3.77	1.73	−0.026	−0.025	0.032	0.206**	–	0.092	0.141	2.96	2.38
6. Argumentativeness	−0.16	1.00	−0.073	−0.132	0.041	−0.043	0.113	–	−0.193**	0.1	0.88
7. Assertiveness	0.12	0.78	−0.146*	−0.074	0.015	0.06	−0.088	−0.142	–	0.43	1.05

*p < 0.05, **p < 0.01.

### Mediation analysis

To verify whether assertiveness and argumentativeness could mediate the effect of the country on the aforementioned independent variance (China = 0, India = 1), we conducted a mediation analysis to examine it, but no significant mediation effect was found in the following results.

*Country – assertiveness – nepotism:* indirect effect = 0.006, bootstrapped 95% CI = [−0.016, 0.018]; *country – assertiveness – relative IOS*: indirect effect = 0.042, bootstrapped 95% CI = [−0.004, 0.029]; *country – assertiveness – relative engaging emotion*: indirect effect = 0.003, bootstrapped 95% CI = [−0.013, 0.015].

*Country – argumentativeness – nepotism:* indirect effect = 0.097, bootstrapped 95% CI = [−0.002, 0.031]; *country – argumentativeness – relative IOS:* indirect effect = 0.056 (6.58% suppressing effect), bootstrapped 95% CI = [0.000, 0.033]; *country – argumentativeness – relative engaging emotion*: indirect effect = −0.010, bootstrapped 95% CI = [−0.020, 0.007].

## Discussion

The results showed that the Chinese felt closer to family members, experienced more engaging emotions, and were more in-group favorite than Indians. Meanwhile, Sino-US differences in these three variables also showed their cultural validities. Moreover, the Chinese were less assertive and argumentative than the Indians. Taken together, it confirmed that the Chinese were more collectivistic than the Indians. However, neither assertiveness nor argumentation could mediate the relationship between the nation (China vs. India) and collectivism, which did not support our mediation hypothesis.

### General discussion

In studies 1 and 2, the measurements of compatriotism, familism, nepotism, cognition style, IOS, and the intensity of experiencing engaging vs. disengaging emotions of Chinese versus Indian participants revealed that the Chinese participants had stronger in-group favoritism in social relations, more holistic thoughts in cognition style, and a more interdependent self in self-construal. Equally important, it also was found that the Chinese are less assertive and argumentative than the Indians in our study. Generally, it is regarded as the characteristic of the individual culture that people are more assertive and argumentative. These findings support the hypothesis that Chinese people are more collectivistic than Indians. The results are also in line with the previous findings of Hofstede (2001), Oyserman et al. (2002a), and Van de Vliert (2011). Moreover, not only self-report methods but also scenario tasks were used here, which made the results more robust.

Unfortunately, the mediation effect of assertiveness or argumentativeness was not supported. It also deserves to be discussed in detail. Lu’s study (2020) suggested that argumentation tradition might contribute to EA-SA (East Asian–South Asian) cultural differences and found that the assertiveness is one mediator between the country (EA vs. SA) and the opportunities for success in the leadership position. The participants of Lu’s study were mainly EA and SA immigrants in the United States. In theory, most of them also are at the elite level in their origin society. However, for other comprehensive samples, the path from cultural heritage concerning argumentation to psychological and behavioral manifestations may be more multiple. In addition, the mediation effect may be more apparent if the study had overcome its disadvantages. For example, our convenience samples include very few participants who are not well-educated or cannot use the Internet tools easily, which may have mitigated the effect to the extent that the mediation was covered.

The societal-level distal culture could cause cognitive, affective, and behavioral consequences through multiple and complex approaches. The mediators include the individual-level internalized cultural values, social institution-level proximal culture, social situation, and subject construal of the situation (Oyserman et al., 2002b). Therefore, other more valid variables concerning argumentation traditions and measures could be used to explore their associations. So far, other well-known ecology factors, such as modernity, paddy rice planting, and the climato-economic hypothesis, could not explain the results. On the whole, it remains to be explained why the Chinese are more collectivistic than the Indians. It will extend our knowledge of the ecology of IND-COL if the antecedents of Sino-India collectivistic differences are verified.

### Theoretical contributions and practical implications

Our findings contributed to the IND-COL differences between China and India. Both China and India are traditional rice-farming, collectivist, Asian countries. They also are representative countries from different culture clusters (Southern Asia and Confucian Asia). A direct comparison between the two broadened the research approach in cultural psychology, and it echoed the calling of IND-COL differences beyond East–West comparison. Although they have been included in the worldwide survey to explore the relationship of IND-COL and other antecedents, outcomes, or as a contrast group of western societies independently, seldom empirical comparison has been made between China and India. We found that the Chinese are more collectivistic than Indians with multiple measures in two convenient independent samples. Meanwhile, we also found that Indians showed an individualistic orientation similar to the Americans in some tasks. For instance, Indian participants felt similar socially engaging emotions as the participants from the United States, which showed independent social orientation. It suggested us being cautious about the generalization of East–West differences to other non-individualistic cultures, which has been mentioned by other cultural psychologists (Van de Vliert, 2020). It means we can differentiate the psychological and cultural characteristics from a smaller range beyond the “West vs. East” frame.

Besides, the Sino-India comparison also has practical implications. Both countries are currently leaders in terms of population, development speed, and economic volume. As such, the need to communicate and cooperate with Chinese people and Indians in all fields is rising fast. A good understanding of them is a prerequisite to good communication and cooperation. Understanding their psychological and behavioral characteristics and cultural differences is essential for their and others’ benefit in business and cultural activities.

Na et al. (2020) found that IND-COL facets, measured by multiple cultural tasks, are not correlated at the individual level in the US and Japan samples. In our studies, only weak or insignificant correlations were found. The results provide further empirical evidence for IND-COL with a loosely connected behavioral profile in India and China. It lends support to IND-COL with a loosely connected profile in more nations.

### Limitations and suggestions for further exploration

First, IND–COL is a multifaceted behavioral synthesis (Triandis, 1995; Na et al., 2020). Only parts of the dimensions of IND–COL were measured, and self-report scales were used in this study. Future studies could measure collectivism from other dimensions and try measuring the variance behaviorally. Second, the surveys were conducted only in English in India. It may make the survey more valid if it is conducted in Hindi in the future.

## Conclusion

It was found that the Chinese are more patriotic, familistic, in-group favorite, interdependent, and holistic than the Indians; meanwhile, they are less assertive and argumentative than the Indians. In brief, the Chinese are more collectivistic than the Indians.

## Data availability statement

The original contributions presented in the study are included in the article/Supplementary material, further inquiries can be directed to the corresponding author.

## Ethics statement

The studies involving human participants were reviewed and approved by the Institutional Review Board of the Institute of Psychology, Chinese Academy of Sciences. The patients/participants provided their written informed consent to participate in this study.

## Author contributions

XR designed the research. DK collected and analyzed the data. XR and DK wrote the manuscript. All authors contributed to the article and approved the submitted version.

## Funding

This work was supported by the National Social Science Fund of China (grant no. 20BSH142) and the Strategic Priority Research Program of Chinese Academy of Sciences (grant no. XDA27000000).

## Conflict of interest

The authors declare that the research was conducted in the absence of any commercial or financial relationships that could be construed as a potential conflict of interest.

## Publisher’s note

All claims expressed in this article are solely those of the authors and do not necessarily represent those of their affiliated organizations, or those of the publisher, the editors and the reviewers. Any product that may be evaluated in this article, or claim that may be made by its manufacturer, is not guaranteed or endorsed by the publisher.
