# Supplementary material for: Sino-India difference in collectivism and its association with cultural heritage concerning argumentation
Source: Front Psychol. 2023 Jan 12;13:1027599. doi: 10.3389/fpsyg.2022.1027599 (PMC9879659; doi:10.3389/fpsyg.2022.1027599)
Supplement: Supplementary file 1 [file Presentation_1.pdf]

Supplementary Material

The full text for contest

Purpose:You are invited to join in a scientific research study on people's thinking mode and related culture.This survey will ask about your thoughts,feelings,motivations etc., It will take your 10 minutes to complete that.

Ethics:You are under no obligation in this survey.If you agree to participate ,you are free to stop your participation at any time by closing the browser window.Your data will then not be used.If any questions that you do not want to answer,just skip to the next question.

Risk:Participating in this study involves no foreseen risks.

Confidentiality:All the information which we had collected from you will be used only for the research,will not be used for other purpose.

you can contact us at *research-study@outlook.com* for any query or doubt.

Thanks for your participation and contribution!
"I have read the above information and agree to participate in this study.I understand that my participation is entirely voluntary and that i may withdraw at any time by closing the browser window."

* 1. **Do you agree to participate in this study(and confirm that you are older than 18)?**

◯ Yes,I agree to participate.

The research data can be accessed by the following linker.

https://www.scidb.cn/en/s/iYvuEf
